# Supplementary material for: MissenseHMM: state-based annotations for missense variants through joint modeling of pathogenicity scores
Source: bioRxiv. 2026 Feb 3:2026.01.31.703062. Preprint. [Version 1] doi: 10.64898/2026.01.31.703062 (PMC12889460; doi:10.64898/2026.01.31.703062)
Supplement: Supplement 1 [file media-1.pdf]

Supplementary Figures

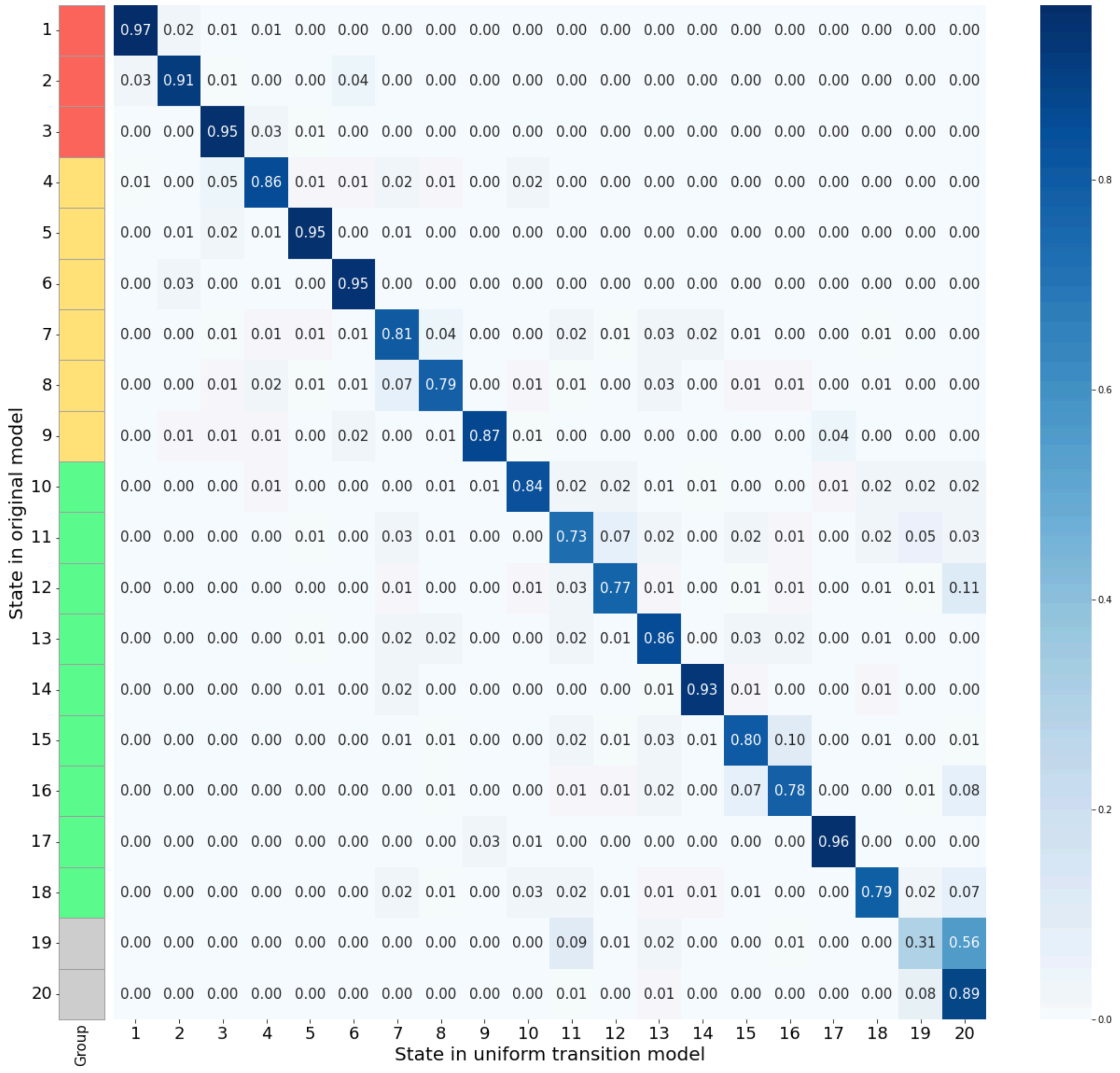

Figure S1: Comparison between the original 20-state model and the uniform transition model. For each state in the original model, the portion of variants assigned to each of the states in the uniform transition model are shown.

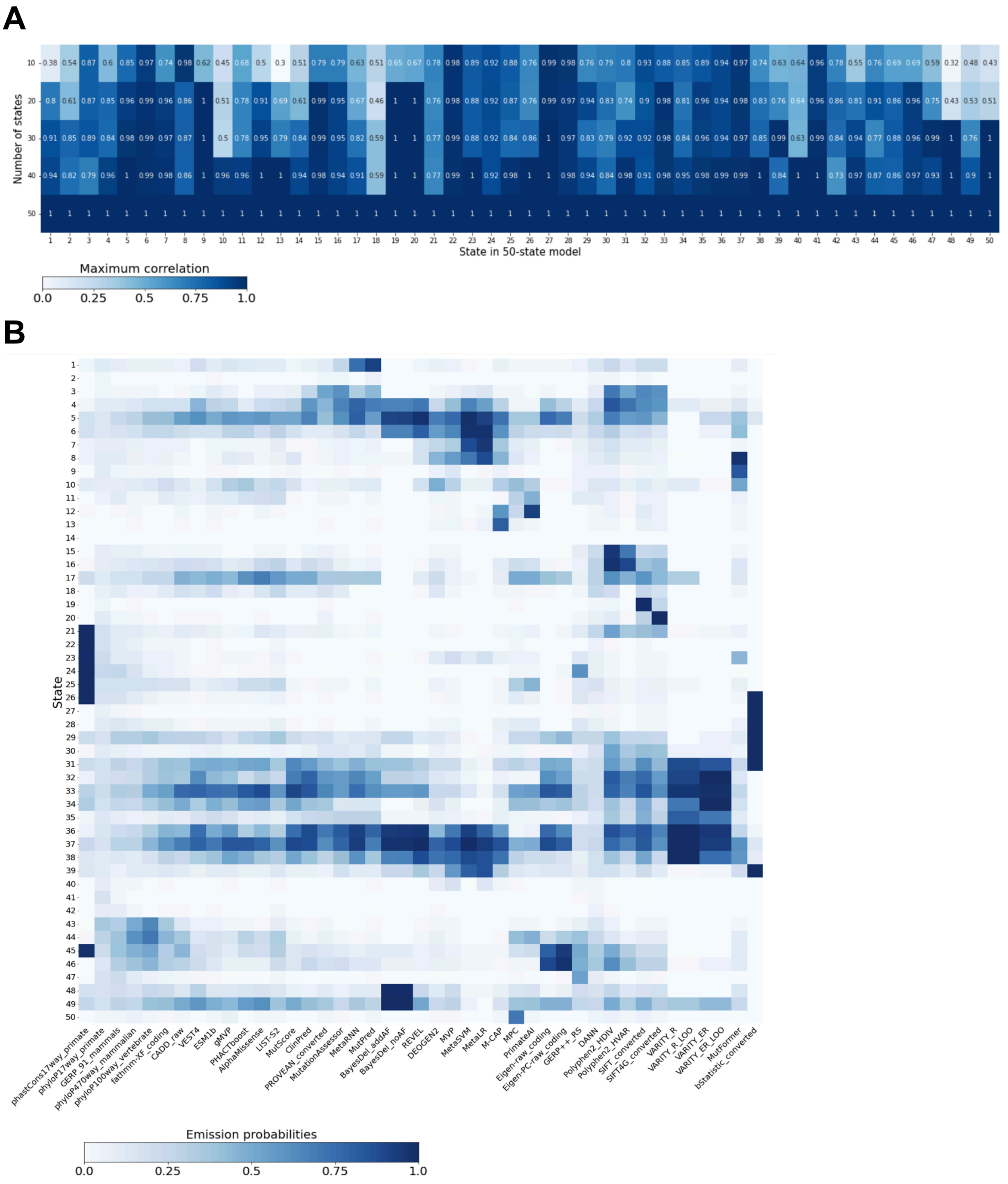

Figure S2 CompareModels results using the 50-state model as reference. (A) Each column represents a state in the 50-state model, and each row corresponds to a model with a different number of states. Heatmap cells indicate the maximum correlation between each state in the 50-state model and its best-matching counterpart in the smaller model. **(B)** Emission parameters for the 50-state model.

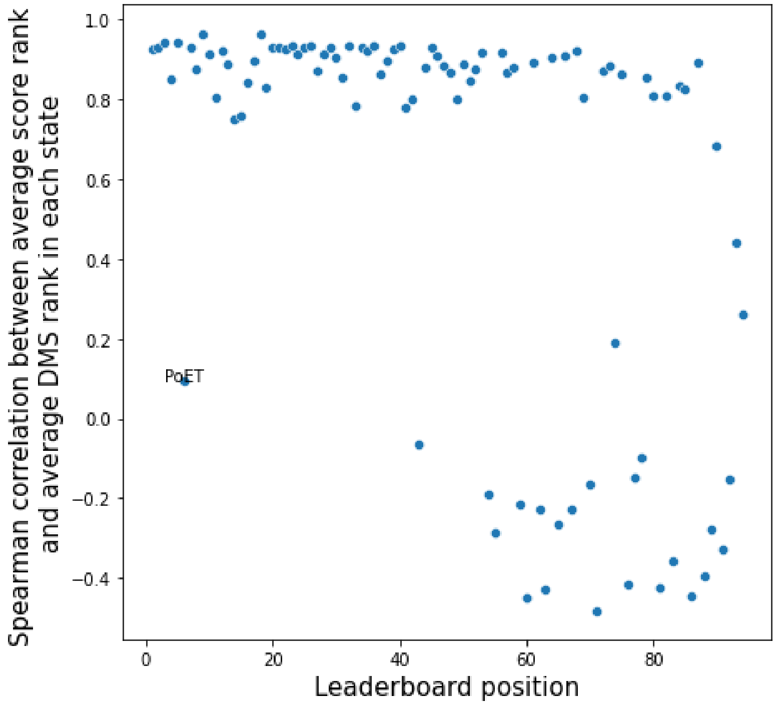

Figure S3: Across-state correlations between predictor scores and DMS measurements. X-axis shows the predictor’s leaderboard positions, with 1 being the highest position. Y-axis shows the Spearman correlation between the predictor’s average score rank in each state and the average DMS rank in each state (Methods).
